# Supplementary material for: The State of Education and Training for Antimicrobial Stewardship Programs in Indian Hospitals―A Qualitative and Quantitative Assessment
Source: Antibiotics (Basel). 2019 Jan 30;8(1):11. doi: 10.3390/antibiotics8010011 (PMC6466562; doi:10.3390/antibiotics8010011)
Supplement: Supplementary file 1 [file antibiotics-08-00011-s001.zip › Supplementary file S2.docx]

**Organisational level:**

**Roles and responsibilities**

1. Do you think you have a role or responsibility in antibiotic prescribing in your organisation?
   1. Why do you think that?
2. Are there any **policies or guidelines** in your organisation for antibiotic prescribing?
3. **What or who influences** how you rank your priorities?
   1. **What influences how you allocate the available resources both human and economic?**
4. At organisational level, what groups of healthcare professionals are identified as having a **role in antibiotic prescribing** activities?

**Education and training:**

1. Do you think there is sufficient teaching on
   1. infection diagnosis and in undergraduate medical curriculum
   2. antibiotic prescribing in undergraduate medical curriculum?
2. Do you think there is sufficient teaching on
   1. infection diagnosis at postgraduate level for doctors
   2. antibiotic prescribing at postgraduate level for doctors?
3. In your healthcare organisation, do healthcare professionals **get on the job, protected training/teaching time**?
4. Is there any **formal teaching** or training programmes in diagnosis of infection and antibiotic prescribing in your organisation?

If yes,

- 1. What professionals are included in this training?
  2. Is it compulsory for staff to attend this training?
  3. How often are staff expected to attend or receive training in this subject?
  4. Do they receive a certificate or qualification for completing the teaching?
  5. Are they assessed as part of the training or teaching?
  6. How is the teaching provided?

1. Do you have a **role or responsibility** in providing **teaching and training** in infection diagnosis and antibiotic prescribing to healthcare professionals?

If yes:

- 1. What professional groups do you teach or train?
  2. How often do you provide teaching or training to these groups?
  3. In your opinion, what are the key necessary topics that need to be covered in this teaching or training?
  4. How do you provide the teaching or training?
  5. What education or training have you had to support your teaching or training?

1. Are you aware of any local, **departmental teaching/training sessions** on infection diagnosis and antibiotic prescribing?
   1. Which departments/specialties do this?
   2. How often?
   3. How is the teaching delivered?
   4. Is it compulsory for staff to attend?
2. In your opinion, is it necessary to provide post-graduate **teaching/training sessions** on infection diagnosis and antibiotic prescribing?
   1. Why?
3. Who do you think should have the responsibility to provide this teaching?
   1. Why?
4. Do you think teaching provided has an impact on antibiotic prescribing behaviours of individual doctors?
   1. Why? How?
5. Do you think teaching provided has had an impact on antibiotic prescribing behaviours of individual doctors in your institution?
   1. Why? How?

At the end:

**How do you think India is doing compared to other similar healthcare systems in relation to AMR/HCAI?**

**In an ideal world what would you like to see implemented on antibiotic prescribing?**

**What are the barriers to implement this?**
