# Supplementary material for: The State of Education and Training for Antimicrobial Stewardship Programs in Indian Hospitals―A Qualitative and Quantitative Assessment
Source: Antibiotics (Basel). 2019 Jan 30;8(1):11. doi: 10.3390/antibiotics8010011 (PMC6466562; doi:10.3390/antibiotics8010011)
Supplement: Supplementary file 1 [file antibiotics-08-00011-s001.zip › Supplementary file S1.docx]

**FCO QUESTIONNAIRE**

**INTRODUCTION**

**…………………………………**

**WORK DETAILS**

1. In which region do you work? (Choose from list)
2. What type of organisation is your main place of work? (Choose from list)
   1. Public or Governmental Hospital
   2. Private Hospital
   3. Community care or clinic
   4. Pharmacy or dispensary
   5. Clinical scientist
   6. Government department
   7. Other (please specify)
3. What specialty do you work in? (Choose from list)
   1. Infectious diseases
   2. Microbiology
   3. Pharmacy
   4. Public health
   5. Healthcare epidemiology
   6. Healthcare admin
   7. Infection prevention‐control
   8. Non-infection specialist clinician (e.g. intensivist, surgeon, internist, general practitioner)
   9. Other (please specify)

**PERSONAL ROLE EDUCATION AND TRAINING**

1. What is your profession? (Choose from list)
   1. Doctor
   2. Pharmacist
   3. Nurse
   4. Non clinical –please specify
   5. Other (please specify)
2. As part of your job, do you do any of the following in relation to antimicrobials: (Choose as many as necessary)
   1. Prescribe
   2. Administer
   3. Review
   4. Teach about infection diagnosis and treatment
   5. Develop antimicrobial prescribing policy and guidelines

**PERSONAL EDUCATION AND TRAINING**

1. Have you received under-graduate education or training in infection management or antimicrobial prescribing?

*Yes/ No/ Not relevant as non-clinical / Not Sure*

1. Have you received post-graduate education or training in infection management or antimicrobial prescribing?

*Yes/No /Not relevant as non-clinical / Not Sure* (if yes go to Q8 If no go to Q11)

1. Who was the training provided by? Choose multiple options as necessary
   1. Employing hospital
   2. Another hospital
   3. University or college
   4. Professional organisation e.g professional society
   5. Private healthcare company
   6. Pharmaceutical company
   7. National or regional governmental agency
   8. Conference
   9. Other
2. Was the training accredited by an external organisation?

*Yes/No/ Not sure (if yes go to Q10 if no go to Q11)*

1. Course Title, accredited by whom (free text)

**YOUR INSTITUTION / ORGANISATION**

1. In your institution or organisation is there a committee or group dedicated to the following: (Options for all- *Yes/No/ Unsure*)
   1. Infection Control and Antimicrobial Stewardship as a single group
   2. Infection Control distinct from Antimicrobial Stewardship
   3. Antimicrobial Stewardship distinct from Infection Control
2. Does your institution or organisation have a formal antimicrobial stewardship programme?

*Yes/No/Not Sure*

1. In your organisation what groups of healthcare professionals are identified as having a role in antimicrobial stewardship?
   1. Nurses
   2. Pharmacists
   3. Doctors
   4. Other (Please specify)

**ORGANISATIONAL EDUCATION AND TRAINING**

1. In your institution or organisation is there a formal strategy or framework for developing and delivering education and training in antimicrobial stewardship or infection control?

*Yes/ No/ Unsure*

1. Do healthcare workers at your institution or organisation receive education or training in antimicrobial stewardship or infection control **at induction** (within three months of starting their job)?

Yes/No/Unsure (if yes go to Q16 if no go to Q18)

1. Which of the following topics are covered in antimicrobial stewardship or infection control education **at induction**? (Choose as many options as necessary)
2. Minimise unnecessary prescribing of antimicrobials
3. Ensure adequate timing of antimicrobial administration
4. Adopt necessary infection prevention and control measures
5. Intravenous administration only in severely ill and/or unable to tolerate oral treatment
6. Obtain biological samples for microscopy, culture and sensitivity testing
7. Review micro results daily, deescalate to narrow-spectrum treatment promptly
8. Review intravenous treatment daily, switch to oral route promptly
9. Therapeutic drug monitoring, following adequate and/or adjusted dosing
10. Require single dose surgical prophylaxis regimens as appropriate
11. Others (please specify)
12. What methods for education and training in antibiotic stewardship or infection control are used at **induction (within 3 months of starting their job)?** (Choose any many options as necessary)
    1. Face-to-face lectures/presentation
    2. Face-to-face workshops or seminars
    3. Work-placed teaching e.g. workbooks or portfolios of evidence
    4. ‘On the job’ learning or learning from practice
    5. Web-based or e-learning
    6. Mixed methods e.g. e-learning and lectures
    7. Don’t educate
    8. Don’t know
    9. Other (please specify)
13. Do healthcare workers at your institution or organisation receive education and training on antimicrobials stewardship **throughout their employment**?

Yes/ No/ Not sure (if yes go to Q19, if no go to Q21)

1. Which of the following topics are covered in antimicrobial stewardship or infection control education **during employment**? (Choose as many options as necessary)
2. Minimise unnecessary prescribing of antimicrobials
3. Ensure adequate timing of antimicrobial administration
4. Adopt necessary infection prevention and control measures
5. Intravenous administration only in severely ill and/or unable to tolerate oral treatment
6. Obtain biological samples for microscopy, culture and sensitivity testing
7. Review micro results daily, deescalate to narrow-spectrum treatment promptly
8. Review intravenous treatment daily, switch to oral route promptly
9. Therapeutic drug monitoring, following adequate and/or adjusted dosing
10. Require single dose surgical prophylaxis regimens as appropriate
11. Others (please specify)
12. What methods of antimicrobial stewardship education or training are for used **throughout employment**? (Choose as many options as necessary)
    1. Face-to-face lectures/presentations
    2. Face-to-face workshops or seminars
    3. Work-placed teaching e.g. workbooks or portfolios of evidence
    4. ‘On the job’ learning or learning from practice
    5. Web-based or e-learning
    6. Mixed methods e.g. e-learning and lectures
    7. Don’t educate
    8. Don’t know
    9. Other (please specify)
13. How frequently is this education or training provided?
    1. Annually or more frequently
    2. Every one to two years
    3. Every two to five years
    4. Ad hoc
    5. Other
14. Is the education or training that is provided at induction mandatory?
15. Is the education or training that is provided during employment mandatory?

*Yes/No/Not Sure*

1. Is attendance at this education or training formally recorded?

*Yes/No/Not Sure*

1. Rate the following statements on a scale of 1-5 with 5 being your preferred option.

I would prefer antimicrobial stewardship education and training to be delivered in:

- Isolation as a single topic
- As part of infection prevention/control education or training
- As part of public health education or training
- As part of patient safety/quality improvement education or training

**LEARNING METHODS AND NEEDS**

1. What are your preferred methods of receiving post-graduate clinical education and training in antimicrobial stewardship and prescribing? Scale of 1-5 with 5 your preferred method.
   1. Face-to-face lectures
   2. Face-to-face workshops or seminars
   3. Work-placed teaching e.g. workbooks or portfolios of evidence
   4. ‘On the job’ learning or learning from practice
   5. Web-based or e-learning
   6. Articles and books
   7. Mixed methods e.g face-to-face lectures and web-based/ e-learning
2. Who do you think should be responsible for providing this education and training? Scale of 1-5 with 5 having the greatest responsibility.
   1. Employing hospital
   2. Employer’s on responsibility/commitment to be up to date
   3. Another hospital
   4. University or college
   5. Professional organisations e.g. professional society
   6. Private healthcare company
   7. Pharmaceutical industry- e.g drug or diagnostics company
   8. National or regional governmental agency
   9. Conference organisers
   10. Other (please specify)
3. Do you think measuring staff participation in education activity should be a metric/indicator for antimicrobial stewardship?

*Yes/ No/ Unsure*

1. Please add up to 2 key observations or comments here in relation to the development, delivery or evaluation of education/training in relation to stewardship.

**THANK YOU AND ROUND-UP**
