# Supplementary material for: The State of Education and Training for Antimicrobial Stewardship Programs in Indian Hospitals―A Qualitative and Quantitative Assessment
Source: Antibiotics (Basel). 2019 Jan 30;8(1):11. doi: 10.3390/antibiotics8010011 (PMC6466562; doi:10.3390/antibiotics8010011)
Supplement: Supplementary file 1 [file antibiotics-08-00011-s001.zip › Supplementary file S3.docx]

| Participant Gender | Occupation | Type of Organisation | Number of beds |
| --- | --- | --- | --- |
| Male | Chair of Infection prevention and Control Committee, Anaesthesiologist | Private | 250 -500 |
| Male | Head of Department Intensive Care Unit, Anaesthesiologist | Private | Less than 250 |
| Male | Head of Department, Infectious Diseases Physician | Private | 250 -500 |
| Female | Head of Department, Medical Microbiologist | Public | 1000+ |
| Female | Head of Department, Medical Microbiologist | Private | 250 -500 |
| Male | Head of Department, Medical Microbiologist | Private | 250 -500 |
| Female | Head of Department, Medical Microbiologist | Private | 250 -500 |
| Female | Head of Department, Medical Microbiologist | Private | 1000+ |
